# Potential of PINK1 and PARKIN Proteins as Biomarkers for Active Multiple Sclerosis: A Japanese Cohort Study

**DOI:** 10.3389/fimmu.2021.681386

**Published:** 2021-08-04

**Authors:** Davide Cossu, Kazumasa Yokoyama, Leonardo Antonio Sechi, Nobutaka Hattori

**Affiliations:** ^1^Department of Neurology, Juntendo University, Tokyo, Japan; ^2^Department of Biomedical Sciences, Sassari University, Sassari, Italy; ^3^SC Microbiologia AOU Sassari, Sassari, Italy

**Keywords:** mitophagy, multiple sclerosis, neuromyelitis optica spectrum disorders, myelin oligodendrocytes glycoprotein (MOG) antibody disorders, PINK1, PARKIN

## Abstract

**Background:**

Mitochondrial dysfunction has been suggested to play an important role in all stages of multiple sclerosis (MS).

**Objective:**

To determine the expression of two mitophagy-related proteins, PTEN-induced kinase 1 (PINK1) and PARKIN, in a cohort of Japanese patients with different neuroinflammatory disorders.

**Methods:**

Protein concentrations were measured using commercial ELISA in paired cerebrospinal fluid (CSF) and serum samples from patients with multiple sclerosis (MS), neuromyelitis optica spectrum disorders (NMOSD), and myelin oligodendrocyte glycoprotein antibody disorders (MOGAD), and from age- and sex-matched controls.

**Results:**

CSF and serum concentrations of PINK1 were higher in patients with MS than in patients with NMOSD (*p* = 0.004 and *p* < 0.001, respectively), MOGAD (*p* = 0.008 and *p* = 0.011, respectively), and controls (*p* = 0.021 and *p* = 0.002, respectively). CSF and concentrations of PARKIN were elevated in patients with MS in comparison with those in controls (*p* = 0.016 and *p* = 0.05, respectively).

**Conclusions:**

Our study highlighted the importance of mitophagy in MS and suggested the potential application of PINK1 and PARKIN as biomarkers to predict disease activity.

## Introduction

Multiple sclerosis (MS) is a T cell-mediated disease of the central nervous system (CNS) primarily characterized by neuroinflammation (1). According to the Multiple Sclerosis International Federation (MSIF), there are more than 2.8 million MS patients worldwide (35.9 per 100,000 population) (2). While the estimated number of patients with MS in Japan is 5,712 (4 per 100,000 population), the number of MS cases has rapidly increased in recent years (2). The clinical course of MS can follow different patterns over time and it can be classified into relapsing-remitting and progressive phases (1). Following periods of relapsing-remitting disease, disability in patients gradually increases with time into a chronic secondary progressive form, characterized by axonal loss and brain atrophy (1). Chronic inflammation contributes to the degeneration of myelin sheath; however, axonal degeneration may occur independent of acute inflammatory lesions, especially in the progressive forms of MS (3). The status of the innate immune system associated with the different stages of MS is not well understood. Indeed, various hypotheses have been proposed to explain the molecular mechanisms of neurodegeneration in MS (3). Among them, mitochondrial dysfunction has been suggested to play an important role in all stages of the disease (3).

Indeed, changes in mitochondrial function have been associated with the pathogenesis of MS (4) as well as with experimental autoimmune encephalomyelitis (EAE) (5), which involves an animal model of autoimmune inflammatory diseases of the CNS. Nohara et al. had previously reported that GDF-15, a mitochondrial disease biomarker, can be a useful marker of MS progression (6). In another retrospective study, Patergnani et al. reported a strong humoral response mounted against PARKIN protein in a cohort of 40 Italian patients with relapsing-remitting MS (RRMS) (7). Furthermore, increased levels of PARKIN in the sera and cerebrospinal fluid (CSF) of gadolinium-positive patients with RRMS indicated that levels of molecular markers of mitophagy are increased in MS patients during the active phases of the disease compared with those in patients without any magnetic resonance imaging (MRI) evidence of disease activity (8).

Parkin is an E3 ubiquitin ligase, encoded by the *PARK 2* gene, that is activated by PTEN-induced kinase 1 (PINK1) on the outer mitochondrial membrane (9). PINK1-PARKIN function together in the pathway of mitochondrial quality control and are mutated in some forms of familial Parkinson’s disease (10). Interestingly, an accumulation of damaged mitochondria may also be related to neuroaxonal damage in NMOSD, an inflammatory CNS disease characterized by optic neuritis and longitudinally extensive myelitis (11).

The role of mitophagy in the pathogenesis of neuroinflammatory and neurodegenerative diseases has remained elusive till date. Considering the heterogeneity in the clinical course of patients with neuroinflammatory and neurodegenerative diseases, we investigated the presence of the specific mitophagy markers, PINK1 and PARKIN, in the bodily fluids of Japanese patients with MS, NMOSD and myelin oligodendrocyte glycoprotein antibody disorders (MOGAD). Our aim is to assess the potential of the two proteins as biomarkers in one or more of these inflammatory diseases.

## Method

### Patients

Serum and CSF paired samples were obtained from 60 Japanese patients recruited at the Juntendo University School of Medicine (Tokyo, Japan). This study was approved by the ethics committee of the Juntendo University School of Medicine (Approval No: 205) in accordance with the Code of Ethics of the World Medical Association (Declaration of Helsinki). All subjects provided written informed consent prior to participation in the study.

The retrospective study included 24 patients with MS diagnosed according to the McDonald criteria (12). The clinical characteristics of patients with MS were as follows: 22 patients were diagnosed with RRMS and two patients with clinically isolated syndrome. In addition, 19 patients diagnosed with NMOSD who fulfilled the 2015 international consensus diagnostic criteria were also enrolled in the study (13). In the NMOSD group, 10 patients presented with an aquaporin 4 (AQP4) antibody-seropositive autoimmune astrocytopathic disease, whereas 9 patients were AQP4-seronegative. The MOGAD group included six patients who tested positive for myelin oligodendrocyte glycoprotein (MOG) antibodies (14). Finally, the control group comprised 11 subjects matched for age and sex from the Juntendo University’s database, with no specific neurological disorders detected at the time of the MRI. The baseline characteristics are presented in **Table 1**.

**Table 1:** Demographic and clinical characteristics of the cohort.

	MS (n = 24)	NMOSD (n = 19)	MOGAD (n = 6)	Controls (n = 11)
**Gender**				
Females n (%)	17 (71)	17 (89)	3 (50)	7 (64)
**Age, years**				
mean ± SD	39 ± 11	44 ± 17	42 ± 16	43 ± 12
**Age at onset, years**				
mean ± SD	31 ± 8	39 ± 17	36 ± 11	43 ± 12
**Disease duration, years**				
mean ± SD	7 ± 6	6 ± 6	6 ± 6	0
**EDSS score**				
median (range)	2 (0–7)	3 (0–7)	3 (0–3.5)	0
**AQP4**				
IgG positive (%)	0 (0)	10 (53)	0 (0)	0 (0)
**MOG**				
IgG positive (%)	0 (0)	0 (0)	6 (100)	0 (0)
**Oligoclonal bands**				
≥ 2 (%)	21 (87)	6 (32)	1 (17)	0 (0)
**IgG index**				
≥ 0.7 (%)	18 (75)	4 (21)	0 (0)	0 (0)
**Albumin quotient**				
≥ 7 × 10^−3^ (%)	6 (25)	6 (32)	0 (0)	0 (0)

MS, multiple sclerosis; NMOSD, neuromyelitis optica spectrum disorder; MOGAD, myelin oligodendrocytes glycoprotein antibody disorders; SD, Standard deviation; AQP4, aquaporin 4; MOG, myelin oligodendrocyte glycoprotein; IgG, Immunoglobulin G.

### Sample Preparation

None of the patients enrolled in the study were undergoing any treatment with immunosuppressants or immune-modulating drugs at the time of blood sampling and lumbar puncture. The CSF was collected through a lumbar puncture as previously reported (15). Within 30 mins following collection, the samples were centrifuged (2000 x g) to remove cells and debris. The samples were then aliquoted and stored at -80°C until analysis. The blood samples were collected in tubes containing a gel separator. Immediately after centrifugation, the sera were stored at -80°C until use. All the CSF and paired serum samples were routinely analyzed for presence of albumin, oligoclonal bands, immunoglobulin G (IgG), IgM, IgA, AQP4-IgG, and MOG-IgG.

### Enzyme-Linked Immunosorbent Assay (ELISA)

CSF and serum levels of native PINK1 and PARKIN proteins were determined using two commercially available ELISA kits (MBS7607221 and MBS732278, respectively; My Biosource; San Diego, California, USA) by following the manufacturer’s instructions. Briefly, PINK1 levels were measured using a sandwich ELISA kit that utilizes a biotin-conjugated anti- PINK1 antibody as the detection antibody. The optical density (OD) measured at the end of the assay was proportional to the concentration of PINK1 protein in the sample captured on the plate. Intensity of the color of the samples was measured spectrophotometrically at 450 nm in a plate reader. In contrast, the levels of PARKIN were assessed using a competitive ELISA kit that utilizes a polyclonal anti-PARKIN antibody and a PARKIN - HRP conjugate. The OD determined at the end of the assay was inversely proportional to the concentration of Parkin, since both the PARKIN protein present in the samples and PARKIN -HRP conjugate competed for the anti- PARKIN antibody binding site. The sample protein concentration was calculated compared with a standard curve that relates the intensity of the sample’s OD to the concentration of the standards Standard curves were plotted by relating the OD to the concentration of PINK1 or PARKIN standards. The protein concentrations in each sample were interpolated from these standard curves. Each standard and sample were assayed in duplicate.

### MRI and Visual Evoked Potential Analyses

Diffusion metrics data were acquired on a 3-T magnetic resonance scanner (Achieva, Philips Medical Systems, Best, The Netherlands) with an 8-channel-array SENSE head coil. After conventional imaging sequences including T2-weighted, T1-weighted, and fluid-attenuated inversion recovery (FLAIR) images were obtained, gadolinium enhancement were occasionally done. Cortical and subcortical lesions were detected by two individual neurologists for blinded fashion with “2D” FLAIR image and occasionally with 3D FLAIR. The visual stimulation protocol entailed a full-field stimulation of each eye with a checkerboard-pattern fixation point. The visual evoked potential parameters recorded were latencies to P100 waves (normal mean, 98.9 ms; upper limit, 110.0 ms). Patients with a P100 latency value greater than 110.0 ms were considered atypical.

### Statistical Analysis

The data were analyzed using GraphPad Prism 9.0 software (GraphPad Software; San Diego, CA, USA). For the multivariate analysis the one-way analysis of variance (ANOVA) with Tukey’s Post-Hoc test was used. Pearson’s correlation coefficient was used to evaluate the correlations between the concentrations of assessed proteins in the sera and CSF of patients. The chi-square test was used to compare categorical variables represented as frequencies. In all analyses, differences were considered significant when *p* ≤ 0.05.

## Results

### CSF and Serum Concentrations of PINK1 and PARKIN

**Figure 1:**
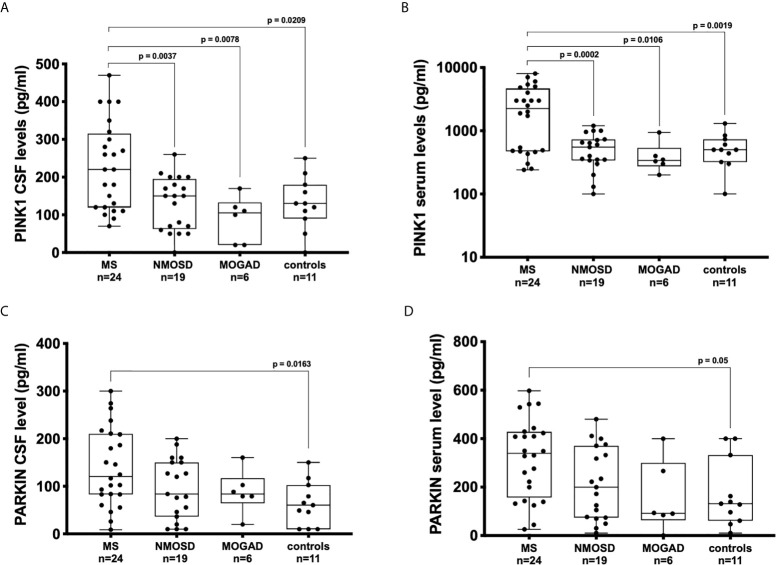
Concentration of PINK1 and PARKIN detected by ELISA in bodily fluids of patients with neuroinflammatory and neurodegenerative diseases and control subjects. Boxplots displaying the medians, interquartile ranges, and total ranges (minimum and maximum) for serum and cerebrospinal fluid (CSF) levels of PINK1 **(A, B)** and PARKIN **(C, D)**. Each dot represents the titer for one patient. ANOVA and Tukey’s multiple comparison test. *P* ≤ 0.05 was considered statistically significant.

The concentrations of PINK1 in the CSF were higher in patients with MS (median: 220, interquartile range: 120-310 pg/mL) than in patients with NMOSD (150, 70-190 pg/mL; *p* = 0.004), MOGAD (110, 20-140 pg/mL; *p* = 0.008), and controls (130, 90-180 pg/mL; *p* = 0.021) (**Figure 1A**).

The concentrations of PINK1 in the sera were higher in patients with MS (median, interquartile range 2250, 480-4520 pg/mL), patients with NMOSD (500, 330-710 pg/mL; *p* < 0.001), MOGAD (340, 270-520 pg/mL; *p* = 0.011), and controls (500, 330-700 pg/mL; *p* = 0.02) (**Figure 1B**).

CSF concentrations of PARKIN were significantly elevated in patients with MS (120, 82-210 pg/mL) in comparison with those in controls (60, 11-103 pg/mL; *p* = 0.016) (**Figure 1C**). Similarly, the serum concentrations of PARKIN were significantly higher in patients with MS (339, 160-425 pg/mL) than in controls (131, 65-330 pg/mL; *p* = 0.05) (**Figure 1D**).

**Figure 2:**
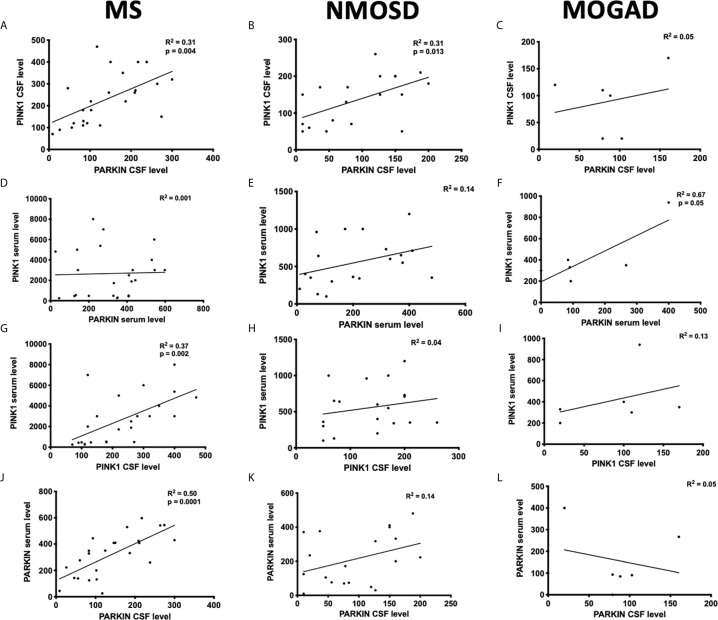
Correlation between PINK1 and PARKIN proteins in the serum and CSF of patients. The distributions represent correlation between the protein concentrations detected in the serum **(A–C)** or CSF **(D–F)** of patients with MS, NMOSD, and MOGAD. Correlation between CSF and serum PINK1 **(G–I)** and PARKIN **(J–L)** in the patients with MS, NMOSD, and MOGAD. Each circle represents the titer for one patient. Pearson’s correlation coefficient. *P* ≤ 0.05 was considered statistically significant.

The regression analysis revealed a significant positive correlation between PINK1 and PARKIN concentrations in the CSF of patients with MS (R^2^ = 0.31; *p* = 0.004), with NMOSD (R^2^ = 0.31; *p* = 0.013) (**Figures 2A, B**), and in the serum of patient with MOGAD (R^2^ = 0.67; *p* = 0.05) (**Figure 2F**). No significant correlation was found between PINK1 and PARKIN levels in the sera of patients with MS and NMOSD (**Figures 2D, E**), as well as in the CSF of MOGAD (**Figure 2C**). Thus, dysfunction of the PINK1-PARKIN pathway may result in a severe impact on neuron and glial cells during neuroinflammation.

A positive correlation was found between serum and CSF concentrations of PINK1 (R^2^ = 0.37; *p* = 0.002) (**Figure 2G**) and PARKIN (R^2^ = 0.50; *p* = 0.0001) (**Figure 2J**) in patients with MS, while no correlation was found in patients with NMOSD and MOGAD (**Figures 2H, I, K, L**).

All patients with RRMS with protein concentration in the serum and CSF above the median (50^th^ percentile) were in the acute phase of the disease with oligoclonal bands positive, high IgG index (≥ 0.7), a high albumin quotient (≥ 7 × 10^−3^), and with high-level baseline Expanded Disability Status Scale) (EDSS) score (**Table 1**). Assessment of the frequency of MRI abnormalities in RRMS patients with higher protein concentrations showed that all patients presented with demyelinating lesions in the cerebral cortex and subcortical areas. Furthermore, a high proportion of subjects presented with lesions in the brainstem (90*%*), cerebellar hemisphere (60*%*), and the spinal cord (50*%*). Mild atrophy in the brain and in the upper cervical spinal cord were seen in 90*%* and 10*%* of the patients with RRMS, respectively. Elevated levels of proteins were detected in patients with NMOSD presented with lesions in the spinal cord (60%), cerebral cortex (30%), brainstem (30%) and optic nerve (30%), while all patients with MOGAD presented with bilateral optic nerve lesions.

Among patients with MS, females exhibited higher concentration to PINK1 and PARKIN than males, with a 2:1 sex ratio. The concentrations of PINK1 were higher in female than male in the CSF (mean: 270 ± 120 pg/mL *versus* 157 ± 92 pg/mL, *p* = 0.04) and in the serum (mean: 3551 ± 2384 pg/mL *versus* 1370 ± 1260 pg/mL, *p* = 0.03); as well as the concentrations of PARKIN in the CSF (mean: 170 ± 81 pg/mL *versus* 87 ± 42 pg/mL, *p* = 0.02) and serum (mean: 373 ± 141 pg/mL *versus* 218 ± 170 pg/mL, *p* = 0.03).

This sex difference could be due to hormonal modulation, environmental, chromosomal influence, and mitochondrial metabolic dysfunctions. Mitophagy is associated with brain metabolism, and there exists sex differences in brain metabolism, as males predominantly utilize proteins while females predominantly use lipids as fuel for mitochondrial energy production (16). Hence, these differences may significantly affect cellular survival following CNS injury.

Finally, no significant difference between AQP4-IgG seropositive and seronegative patients with NMOSD were observed linked to the concentration of the two proteins.

## Discussion

Mitochondrial dysfunctions are common in MS and classical neurodegenerative diseases such as Alzheimer’s and Parkinson’s diseases, which have different physiopathology in their onset, but they have a similar course of gradual and progressive neuronal loss (17). However, the role of mitophagy in the pathogenesis of neuroinflammatory and neurodegenerative diseases has not been examined in previous studies.

In this study, we reported a statistically significant increase in the levels of PARKIN and PINK1 in the CSF and paired serum samples of patients with RRMS in the acute phase, highlighting the importance of mitophagy in the etiopathogenesis and progression of MS. The impairment of mitochondrial energy metabolism can lead to increased reactive oxygen species, loss of Ca^2+^ homeostasis, and necrotic or apoptotic cell death, culminating in the hyperactivation of inflammatory signaling pathways and eventually chronic systemic inflammation (18). In demyelinating diseases such as MS, these processes are aggravated because the energy-intensive axonal area significantly increases (19), whereas in patients with autosomal recessive familial Parkinson’s diseases resulting due to mutation in the *Parkin* gene, oxidative stress plays an important role in the degeneration of dopaminergic neurons (20).

Our data were in agreement with those in previous studies, where increased levels of PARKIN in the bodily fluids of patients with RRMS in the acute phase were detected compared with those in controls (7, 8, 21, 22). For the analysis of PARKIN concentration, we used the same ELISA kit that was used in previous studies, as competitive ELISAs are fast and have a good reproducibility. However, we used a more sensitive sandwich ELISA for the detection of PINK1 because this analysis was done for the first time. Overall, both procedures provided a sensitive and specific methods for the detection of PARKIN and PINK1 proteins, indicating a potential use as a screening procedure in the setting of acute MS.

The mean serum and CSF concentration of Parkin in our MS Japanese group were higher than values found in the Italian and Indian MS cohort (7, 8, 21, 22), suggesting that MS patients from different ethnicity and genetic backgrounds have altered immunopathogenesis (23).

The presence of higher serum concentration of the PINK1 and PARKIN proteins may be explained with the “outside-in” theory, wherein an immune response is initiated in the periphery and progresses into the CNS (24). Since CSF and serum PINK1 and PARKIN levels were highly correlated in RRMS patients with elevated albumin quotient, we can speculate that protein concentration raised due to an increased permeability of the blood-brain barrier.

However, it should be noted that the activation of an autoimmune response may also represent a secondary reaction to a primary degenerative process occurring in the CNS (24).

The higher PINK1/PARKIN concentration found in the serum compared to the CSF, could be due to the exosomal cross talk from the periphery and within the CNS (25). Brain-derived exosomes can efflux and go to the blood through the brain lymphatic drainage system or crossing the blood-brain barrier (25), thereby altering the protein-trafficking.

Additionally, we also reported, for the first time, augmented concentrations of PINK1 protein in patients with RRMS in the acute phase compared with those in patients with NMOSD and MOGAD. Interestingly, a study reported distinct expression of PINK1 using immunostaining in astrocytes in active demyelinating MS lesions, whereas astrocytes in chronic lesions were weakly stained (26).

Along with their role in mitophagy, PINK1 and PARKIN proteins also play an important role in the development of astrocytes (27). Mitochondrial dysfunction may impair the functionality of astrocytes, causing reactive gliosis that eventually leads to the formation of the glial scar (28). Astrocytes are considered an important indicator of lesion activity and age (29). Consequently, the presence of these mitophagy markers in the CSF of patients with RRMS may also be linked to a decline in normal astrocytic functions.

The main limitations of this study include the limited number of subjects enrolled and the primary inclusion of patients with RRMS in the MS patient cohort. While RRMS is the most common type of MS, representing between 85 % − 90% of cases, it is well known that mitophagy activity declines with aging (30). Older patients are more likely to have a secondary progressive MS diagnosis than young patients (31). As a result of this limitation, we were unable to report on the presence of mitophagy marker expression in other forms of MS. Despite the challenging diagnosis of the conversion from RRMS to secondary progressive MS, often diagnosed retrospectively, further follow-up studies are required to monitor the concentration of these markers with respect to advancing age or the clinical course of the disease. Finally, knockout animal models may be useful for understanding the precise role of PINK1 and PARKIN role in the context of neuroinflammation.

In conclusion, our study confirmed the role of mitophagy in the pathogenesis of MS, suggesting for the first time a direct relationship of PINK1 expression to RRMS, but not NMOSD and MOGAD. Since blood is a highly accessible bodily fluid compared with CSF, additional research is needed to clarify to what extent (e.g., concentration of protein) and under which conditions (e.g., comorbidities such as diabetes or other metabolic alterations) serum levels of PARKIN and especially PINK1 can serve as biomarkers capable of distinguishing patients with RRMS in the acute phase from patients with other neurological disorders such as NMOSD or MOGAD.

## Data Availability Statement

The raw data supporting the conclusions of this article will be made available by the authors, without undue reservation.

## Ethics Statement

The studies involving human participants were reviewed and approved by the ethics committee of the Juntendo University School of Medicine (Approval No: 205) in accordance with the Code of Ethics of the World Medical Association (Declaration of Helsinki). The patients/participants provided their written informed consent to participate in this study.

## Author Contributions 

DC: conceptualization, methodology, data curation, writing-original draft preparation. KY, LS, and NH: supervision and reviewing. All authors contributed to the article and approved the submitted version.

## Funding

This study was supported by JSPS KAKENHI Grant Number: JP 20K16468.

## Conflict of Interest

The authors declare that the research was conducted in the absence of any commercial or financial relationships that could be construed as a potential conflict of interest.

## Publisher’s Note

All claims expressed in this article are solely those of the authors and do not necessarily represent those of their affiliated organizations, or those of the publisher, the editors and the reviewers. Any product that may be evaluated in this article, or claim that may be made by its manufacturer, is not guaranteed or endorsed by the publisher.
